# Evaluation of the effects of postprocessing settings in digital bitewing radiographs on proximal caries detection

**DOI:** 10.1002/cre2.889

**Published:** 2024-05-07

**Authors:** Mehrdad Abdinian, Forooz Keshani, Fateme Sadeghi, Parisa Soltani, Gianrico Spagnuolo, Carlo Rengo

**Affiliations:** ^1^ Department of Oral and Maxillofacial Radiology, Dental Implants Research Center, Dental Research Institute, School of Dentistry Isfahan University of Medical Sciences Isfahan Iran; ^2^ Department of Oral and Maxillofacial Pathology, Dental Research Center, Dental Research Institute, School of Dentistry Isfahan University of Medical Sciences Isfahan Iran; ^3^ Students Research Committee, School of Dentistry Isfahan University of Medical Sciences Isfahan Iran; ^4^ Department of Neurosciences, Reproductive and Odontostomatological Sciences University of Naples “Federico II” Naples Italy; ^5^ Therapeutic Dentistry Department, Institute for Dentistry Sechenov University Moscow Russia

**Keywords:** dental caries, digital radiography, intraoral radiography, radiography

## Abstract

**Objective:**

Radiographs are an integral part of detecting proximal caries. The aim of this study was to evaluate the effect of contrast, brightness, noise, sharpness, and *γ* adjustment of digital intraoral radiographs on the diagnosis of proximal caries.

**Materials and methods:**

In this in vitro study, 40 extracted teeth including 20 premolars and 20 molars with enamel lesions (white spot or dentin discoloration seen through the enamel) were mounted together in groups of eight inside the skull. Bitewing radiographic images of each dental group were obtained by a photostimulable phosphor plate sensor with exposure conditions of 8 mA, 70 kV, and 0.2 s. The images were reconstructed by the built‐in software and examined by two oral and maxillofacial radiologists in various settings of contrast, brightness, sharpness, noise, and *γ*. The teeth were then cut mesiodistally and the presence or absence of caries was confirmed by an oral and maxillofacial pathologist using a stereomicroscope. The data were then analyzed using the *κ* agreement coefficient, sensitivity, specificity, and accuracy (*α* = .05).

**Results:**

Adjustment of brightness and contrast led to higher diagnostic performance with an accuracy of 82.5% and 83.8 (for observers 1 and 2, respectively) and 82.5% (for both observers), respectively. Noise adjustment was the least helpful approach for diagnosis of proximal dental caries among other adjustments, with an accuracy of 78.8% and 77.5% for observers 1 and 2, respectively.

**Conclusion:**

Brightness and contrast setting was more efficient in improving the diagnostic potential of bitewing radiographs compared to other adjustments.

## 1 INTRODUCTION

Interproximal caries occur primarily as opaque spots with reduced enamel translucency in the proximal surface of teeth between the marginal gingiva and interproximal contact (Kamburoglu et al., 2012; Senel et al., 2010). Early detection of interproximal caries is important for preventing the loss of dental structure and pulpoperiapical inflammation. Proximal surfaces are hard to visualize via direct vision clinically and thus, radiographic examinations play an important role in detecting initial carious lesions in these surfaces. However, demineralization of enamel cannot be detected on radiographic examination unless approximately 30%–40% of the mineral substance is lost (Krzyżostaniak et al., 2015). Therefore, accurate diagnosis of proximal caries in bitewing radiographs can be challenging (Lee et al., 2021).

Digital imaging is the prevailing imaging technique in dental practice because it provides many benefits including easy transfer of images, postprocessing options, and lower radiation doses compared to conventional analog imaging (Lo Giudice, Nucera, et al., 2020; Miri et al., 2015). Software used for the acquisition, display, and interpretation of digital images provides a variety of postprocessing options, such as sharpening, zooming, manipulation of brightness, contrast, and *γ*, and noise removal (Lo Giudice, Rustico, et al., 2020). Many studies have been performed to determine the effectiveness of these tools for various diagnostic tasks. Most of these studies have focused on contrast and brightness and few studies have been performed on other tools including noise and *γ* adjustments (Nascimento et al., 2018; Shah et al., 2020). In general, lower brightness and higher contrast are preferred for the detection of proximal caries (Gaêta‐Araujo, 2019; Nascimento et al., 2018). To our knowledge, studies comparing the effects of all major post‐processing options are scarce. Additionally, a more in‐depth analysis of the existing literature shows that most studies focus on in‐built features native to certain radiologic analysis software. For instance, Kajan et al. (2015) investigated the effects of several filters such as sharpness, noise reduction, magnification, and enhancement in Scanora software (version 0.8) and reported that “Sharpening UM” and “Magnification 1:3” filters improved the diagnostic accuracy and the observer agreement more effectively than the other processing tools. Additionally, Shokri et al. (2018) used the same software (version not mentioned) and found that enhancement filter 2 was the superior tool in increasing the accuracy of digital bitewing images. Although these reports provide valuable information, since they have used the predetermined filters by the manufacturers, their replication in other radiologic analysis software may be difficult. Hence, investigating the effects of manual adjustment of different image features can be more generalizable to the existing radiologic analysis software. Therefore, the aim of the present study was to evaluate the effects of contrast, sharpness, brightness, noise, and *γ* adjustments on the detection of interproximal caries in bitewing radiographs.

## 2 METHODS

The protocol of this study was approved by the Research Ethics Committee at Isfahan University of Medical Sciences (#IR.MUI.RESEARCH.REC.1399.757, approval date: 01/03/2021). The samples were teeth extracted for unrelated reasons to this study. This experimental study was performed on premolar and molar teeth with interproximal white spot lesions and enamel discoloration. Sample size (n) was calculated based on the following equation:

$$n=\frac{{\left(Z_{1-\frac{\alpha }{2}}+Z_{1-\beta }\right)}^{2}[p_{1}\left(1-p_{1}\right)+p_{2}\left(1-p_{2}\right)]}{d^{2}}$$

where *α* = .05, *β* = .20, *p*_1_ and *p*_2_ (proportions) = .5 to yield the highest sample size, and *d* (margin of error) = 0.31. Therefore, a minimum of 40 teeth were required.

### 2.1 Preparation of samples

Twenty premolar and 20 molar teeth extracted for reasons such as periodontal involvement and orthodontic purposes were used in this study. The teeth with white spot lesions and enamel discoloration (International Caries Detection and Assessment System [ICDAS] 1 and 2) were selected as well as those without any visible enamel defect (ICDAS 0) (Gugnani & Pandit, 2011). The teeth were evaluated under magnification and those with cavitation of the proximal surfaces were excluded from the study. Debris was removed from the teeth and the teeth were placed in 2% hypochlorite sodium solution for 20 min. The teeth were thereafter kept in normal saline. The crown of the teeth was cut using a diamond bur 2 mm apical to the cemento‐enamel junction. The teeth were then mounted using rose wax in three unidentified human skulls in their corresponding place in half of the maxillary (four teeth) and mandibular (four teeth) arches on each side and the proximal contacts and occlusion with the opposing teeth were recreated. Therefore, a total of five experimental set‐ups were prepared. Thereafter, the mandible and the cranium were secured in occlusion using tape. The soft tissue was simulated using baseplate wax (Polywax, Bilkim Co. Ltd) with a thickness of 12 mm in the buccal surface of the jaws.

### 2.2 Radiographic examination and interpretation

Bitewing radiographs were obtained using photostimulable phosphor plates (Durr Dental) with an intraoral radiographic unit (Planmeca) using exposure parameters of 70 kVp, 8 mA, and 0.2 s exposure time with a distance of 20 cm from the source to object distance. Radiographs were obtained using a film holder (Kerr Dental). The horizontal angulation of the intraoral tube was set parallel to the buccal surface of the teeth and the vertical angulation was +5°. In total, five bitewing radiographs were prepared from the skulls each containing eight teeth.

**Figure 1:**
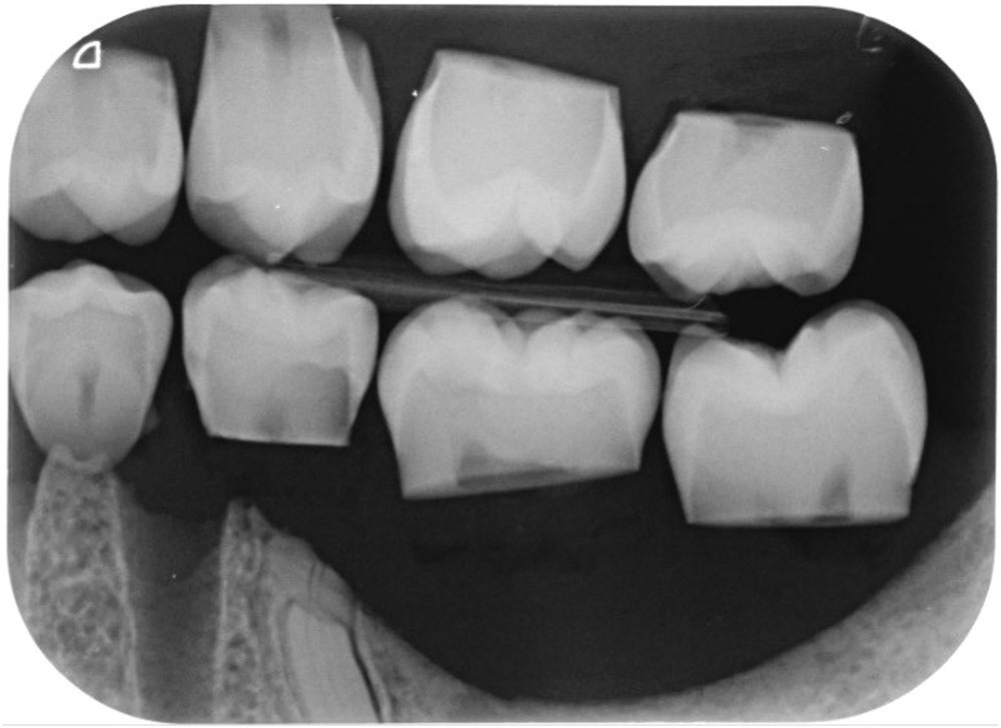
Digital bitewing radiographs of some of the sample teeth.

The radiographic images were viewed in Scanora software (Soredex) by two oral and maxillofacial radiologists (with 5 and 13 years of experience) in a quiet dimly lit room using a monitor (Figure 1). Six viewing sessions were planned for each observer. In the first viewing session, the observers viewed the original radiographic images without altering other parameters. In the other five viewing sessions, the observers were asked to adjust only one of the parameters of sharpness, brightness, contrast, *γ*, and noise in each session, without altering other parameters. For each observer, the viewing sessions were planned at 2‐week intervals. The proximal surface of each tooth was assessed using the following scale: 0: *no caries present*; 1: *proximal caries present*; 2: *uncertain*. A third oral and maxillofacial radiologist was consulted in cases of disagreement. Teeth with uncertain decisions were planned to be excluded. To assess intraobserver agreement, 20% of the radiographic images were viewed by the observers once again after 1 month.

### 2.3 Histopathologic examination

For histopathologic examination, the teeth were sectioned mesiodistally along their longitudinal axis using diamond disks, and sections with appropriate thickness were obtained. Thereafter, the sections were mounted on a slide and viewed by an oral and maxillofacial pathologist (with 8 years of experience) under a stereomicroscope (SMP200, HP). Demineralized lesions or yellow‐brown discolorations were regarded as caries using the following scale: 0: *no caries present*; 1: *proximal caries present*. The histologic results were considered the gold standard against which the radiographic findings were compared.

### 2.4 Statistical analysis

Statistical analysis was performed using Statistical Package for the Social Sciences (SPSS, v. 26, IBM). Intraobserver and interobserver agreements were calculated using Cohen's *κ*. The significance level was set at 0.05. Sensitivity, specificity, and accuracy values for adjustment of sharpness, contrast, brightness, *γ*, and noise were calculated using the following formula:

$$\mathrm{Sensitivity}=\frac{\mathrm{True}\;\mathrm{positive}}{\mathrm{True}\;\mathrm{positive}+\mathrm{False}\;\mathrm{negative}},$$

$$\mathrm{Specificity}=\frac{\mathrm{True}\;\mathrm{negative}}{\mathrm{True}\;\mathrm{negative}+\mathrm{False}\;\mathrm{positive}},$$

$$\mathrm{Accuracy}=\frac{\mathrm{True}\;\mathrm{positive}+\mathrm{True}\;\mathrm{negative}}{\mathrm{True}\;\mathrm{positive}+\mathrm{False}\;\mathrm{negative}+\mathrm{True}\;\mathrm{negative}+\mathrm{False}\;\mathrm{positive}}.$$

## 3 RESULTS

When using different postprocessing settings, intraobserver agreements were excellent (0.85–1.00, *p* < .001), while interobserver agreements ranged between 0.72 and 1.00 (*p* < .001) indicating good and excellent agreement between the observers.

Adjustment of brightness and contrast led to higher diagnostic performance with an accuracy of 82.5% and 83.8 (for observers 1 and 2, respectively) and 82.5% (for both observers), respectively. Noise adjustment was the least helpful approach for diagnosis of proximal dental caries among other adjustments, with an accuracy of 78.8% and 77.5% for observers 1 and 2, respectively (Table 1).

**Table 1:** Sensitivity, specificity, and area under curve for different post‐processing adjustments.

	Adjustment	Sensitivity (%)	Specificity (%)	Accuracy (%)
Observer 1	Original	40.0	90.9	75.0
	Contrast	42.3	93.4	82.5
	Brightness	43.0	93.4	82.5
	Sharpness	36.0	93.4	81.3
	*γ*	29.5	93.4	80.0
	Noise	29.5	91.9	78.8
Observer 2	Original	60.0	90.9	81.3
	Contrast	48.5	93.4	83.8
	Brightness	42.3	93.4	82.5
	Sharpness	29.8	91.9	78.8
	*γ*	36.0	91.9	80.0
	Noise	24.3	91.9	77.5

## 4 DISCUSSION

The present study sought to answer the following question: Which postprocessing adjustment on digital bitewing radiographs was more helpful for the diagnosis of proximal caries? Based on the findings of this study, while adjustment of contrast and brightness were the most effective settings for the diagnosis of proximal caries, noise alteration was the least effective adjustment for this diagnostic task.

In their study in 2015, Kajan et al. (2015) evaluated the effects of noise reduction, sharpening, enhancement, and magnification on the detection of non‐cavitated proximal dental caries. Based on their findings, the application of “Sharpening UM” tool along with the “Magnification 1:3” processing option improved the diagnostic accuracy and the observer agreement more effectively than the other processing procedures (Kajan et al., 2015). However, since clinicians and dental practitioners use a variety of digital radiological analysis software, employing manual modifications instead of fixed software filters will make the findings of research studies more universal with a higher degree of generalizability to different radiological analysis software (Mehdizadeh et al., 2023).

Shokri et al. (2018) investigated the effects of enhancement filters and denoising of digital bitewing radiographs on the detection of proximal and occlusal caries. They concluded that in Scanora radiographic analysis software, enhancement filter 2 with or without denoising was the most effective in improving the diagnosis of dental caries (Shokri et al., 2018). This finding is consistent with our finding that noise adjustment was the least effective option among other postprocessing settings. However, it has to be mentioned that the aim of the two studies was different as the present study sought to find the most effective adjustment for the detection of noncavitated caries regardless of the direction of the setting.

In their study in 2015, Miri et al. (2015) investigated the effects of the reverse contrast technique on the detection of proximal caries in bitewing radiographs. Their findings revealed that radiographic images with reversed contrast were not more accurate compared to the original ones. In fact, the sensitivity of reverse contrast images was significantly less than the original ones (Miri et al., 2015). These findings show the importance of considering the effects of post‐processing adjustments on the accuracy of detection as not all of these image alterations lead to improvements in the diagnosis. In our study, we did not focus on reversing the contrast and instead investigated the effects of increasing or decreasing the contrast which leads to the highest diagnostic values.

In another study by Nascimento et al. (2018), the effects of contrast and brightness alterations of digital intraoral images on the diagnosis of proximal caries lesions were examined. No statistical difference was found between the diagnostic performance of the original and processed radiographic images, while the observers preferred images with lower brightness and higher contrast (Nascimento et al., 2018). This finding highlights the gap between observer preferences and the diagnostic value of radiographic images (Shujaat et al., 2020).

Caries lesions tend to progress slowly and thus, in the diagnosis of dental caries, higher specificity values of a diagnostic tool are more important compared with higher sensitivity values to prevent unnecessary restorative interventions (Hummel et al., 2019; Shokri et al., 2018). Bitewing radiographs, with high specificity, allow for a reduction in misdiagnosis of intact surfaces and unnecessary restorative procedures (Schwendicke & Göstemeyer, 2019). Based on our findings, altering the contrast and brightness would be helpful in this diagnostic task.

One of the limitations of this study is its in vitro design. Simulation of clinical conditions is a challenge in such in vitro studies. However, several measures have been taken, including using wax as a simulant for soft tissue (Schropp et al., 2012) as well as re‐establishing proximal contacts, to replicate the clinical settings more accurately.

## 5 CONCLUSION

For diagnosis of proximal dental caries using digital bitewing radiographs, post‐processing adjustment of contrast and brightness were the most effective settings, while noise alteration was the least effective adjustment for this diagnostic task.

## AUTHOR CONTRIBUTIONS

Mehrdad Abdinian, Fateme Sadeghi, and Parisa Soltani participated in the design and conceptualization of the study. Mehrdad Abdinian, Forooz Keshani, Fateme Sadeghi, and Parisa Soltani participated in the methodology. Carlo Rengo and Gianrico Spagnuolo participated in data interpretation and analysis. Mehrdad Abdinian, Fateme Sadeghi, and Carlo Rengo wrote the initial draft. Forooz Keshani, Parisa Soltani, and Gianrico Spagnuolo edited the draft. All authors have read and approved the final manuscript.

## CONFLICT OF INTEREST STATEMENT

The authors declare no conflict of interest.

## Data Availability

The data that support the findings of this study are available from the corresponding author upon reasonable request.
